# Experimental induction of proventricular dilatation disease in cockatiels (*Nymphicus hollandicus*) inoculated with brain homogenates containing avian bornavirus 4

**DOI:** 10.1186/1743-422X-6-100

**Published:** 2009-07-09

**Authors:** Ady Y Gancz, Amy L Kistler, Alexander L Greninger, Yigal Farnoushi, Sara Mechani, Shmuel Perl, Asaf Berkowitz, Noa Perez, Susan Clubb, Joseph L DeRisi, Don Ganem, Avishai Lublin

**Affiliations:** 1 The Exotic Clinic, Herzlyia, 46875, Israel; 2 Howard Hughes Medical Institute, MD, USA; 3 Department of Biochemistry, Microbiology and Medicine, University of California, San Francisco 94143, USA; 4 Division of Avian & Fish Diseases, Kimron Veterinary Institute, Bet Dagan, 50250, Israel; 5 Department of Pathology, Kimron Veterinary Institute, Bet Dagan, 50250, Israel; 6 Koret School of Veterinary Medicine, The Hebrew University of Jerusalem, Rehovot 76100, Israel; 7 Rainforest Clinic for Birds and Exotics, Loxahatchee, FL 33470, USA

## Abstract

**Background:**

Proventricular dilatation disease (PDD) is a fatal disorder of psittacine birds worldwide. The disease is characterized by lymphoplasmacytic infiltration of the central and peripheral nervous systems, leading to gastrointestinal motility and/or central nervous system dysfunction. Recently, we detected a significant association between avian bornavirus (ABV) infection and clinical signs of PDD in psittacines. However, it remains unclear whether ABV infection actually causes PDD. To address this question, we examined the impact of ABV inoculation on the cockatiel (*Nymphicus hollandicus*).

**Results:**

Five cockatiels were inoculated via multiple routes (intramuscular, intraocular, intranasal, and oral) with a brain homogenate derived from either a PDD(+) avian bornavirus 4 (ABV4) (+) case (n = 3 inoculees) or from a PDD(-) ABV(-) control (n = 2 inoculees). The control birds remained free of clinical or pathological signs of PDD, and tested ABV(-) by RT-PCR and immunohistochemistry (IHC). In contrast, all three cockatiels inoculated with ABV4(+) brain homogenate developed gross and microscopic PDD lesions, and two exhibited overt clinical signs. In numerous tissues, ABV RT-PCR and sequence analysis demonstrated the presence of ABV4 RNA nearly identical to that in the inoculum. ABV was detected in the central nervous system of the three ABV-inoculees by IHC. Pyrosequencing to investigate the viral flora in the ABV4(+) inoculum uncovered 7 unique reads sharing 73–100% nucleotide sequence identity with previously identified ABV sequences and 24 reads sharing 40–89% amino acid sequence identity with viruses in the *Retroviridae *and *Astroviridae *families. Of these candidate viral species, only ABV RNA was recovered from tissues of the inoculated birds.

**Conclusion:**

In this study, the clinical and pathological manifestations of PDD were induced by inoculation of cockatiels with brain homogenates containing avian bornavirus 4. By using high throughput pyrosequencing an in-depth view of the viral content of the inoculum was achieved, revealing that of 3 candidate virus families detected, only the presence of ABV RNA correlated with the development of PDD. This study provides evidence of a causal association between ABV4 infection and PDD in cockatiels.

## Background

Proventricular dilatation disease (PDD) is a fatal inflammatory disease of psittacine birds (parrots), characterized by lymphoplasmacytic infiltration of the central and peripheral nervous systems, leading to gastrointestinal (GI) motility malfunction and/or central nervous system disorders. The disease has been documented in multiple continents and in over 50 different species of psittacines as well as captive and free-ranging species in at least 5 other orders of birds [1-4]. PDD is considered to be among the greatest threats to aviculture of psittacines, including several highly endangered species.

PDD primarily affects the autonomic nerves of the upper and middle digestive tract, including the esophagus, crop, proventriculus, ventriculus, and duodenum. Microscopically, the disease is recognized by the presence of lymphoplasmacytic infiltrates within myenteric ganglia and nerves. Similar infiltrates may also be present in the brain, spinal cord, peripheral nerves, conductive tissue of the heart, and adrenal glands. Non-suppurative leiomyositis and/or myocarditis may accompany the neural lesions [5-8]. Clinically, PDD cases present with GI tract dysfunction (dysphagia, regurgitation, and passage of undigested food in feces), neurologic symptoms (e.g. ataxia, abnormal gait, proprioceptive defects), or both [3]. Although the clinical course of the disease can vary, PDD is generally fatal if left untreated [3].

Since its initial description in the 1970s, a viral etiology for PDD has been suspected; however, for some time, the identity of this agent remained elusive. Recently, we reported the detection of an association between histologically confirmed PDD in psittacines and infection with a novel clade of avian bornaviruses bornaviruses (ABV) [9]. Similar findings involving 2 independent case/control studies have since been reported by others [10,11], offering support to the possibility that ABV may indeed be the etiologic agent of PDD. However, direct evidence that infection with ABV transmits the symptoms and pathology characteristic of PDD has yet to be observed. Here we describe the results of experimental inoculation of cockatiels (*Nymphicus hollandicus*) with brain homogenates containing avian bornavirus 4 (ABV4).

## Results

### Clinical observations and post-mortem macroscopic findings

**Figure 1:**
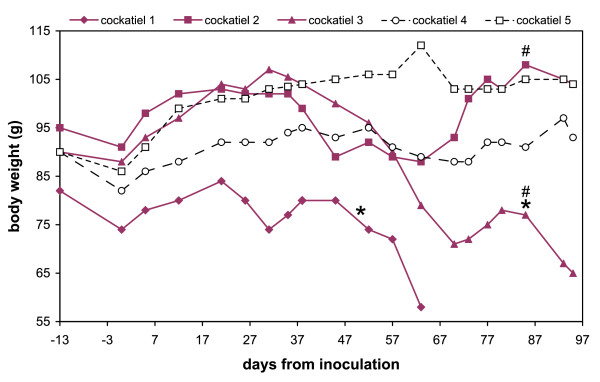
**Body weights of the ABV4-inoculated and sham-inoculated cockatiels during the study period**. The ABV4-inoculated birds (cockatiels 1–3), are shown in bald lines with solid symbols. Cockatiels 4,5 are the control birds; "*" marks the first observation of undigested seeds in the feces; "#" marks the first detection of ABV4 RNA in a choanal or cloacal swab by RT-PCR. Note the continuous decrease in BW of cockatiels 1 and 3 starting on day 21 PI and 31 PI, respectively.

Five cockatiels were each inoculated via multiple routes (intramuscular, intraocular, intranasal, and oral) with a brain homogenate derived from either a PDD(+) ABV(+) bird (n = 3) or from a PDD(-) ABV(-) bird (n = 2). The birds were then followed for 95 days for behavioral and clinical manifestations. Two out of three cockatiels in the ABV-inoculated group developed overt clinical manifestations typical of PDD during the study period. In the first bird to show clinical signs (cockatiel 1), a sharp decrease in body-weight (BW) and body condition was observed starting on day 21 post-inoculation (PI), and undigested seeds were present in the feces from day 50 PI. In the second bird (cockatiel 3) these same clinical signs were observed starting on days 31 and 85 PI, respectively (Figure 1). Interestingly, from day 9 PI onwards, cockatiel 1 had started plucking feathers over its entire trunk. The bird was also reluctant to move around in its cage, as evident by the accumulation of faeces on a single spot underneath its perch. These signs were not observed in any of the other study birds. After losing nearly 30% of its initial BW, and exhibiting signs of severe weakness, cockatiel 1 was humanely euthanized on day 64 PI. The other birds in the study were euthanized at the end of the study period (95 days PI). At this time, cockatiel 3 had lost >25% of its initial BW. The third ABV-inoculated bird (cockatiel 2) and the two control birds (cockatiels 4 and 5) did not show overt clinical signs of PDD during the study period. However, the BW of cockatiel 2 did appear to fluctuate while those of the control birds remained fairly stable (Figure 1).

**Figure 2:**
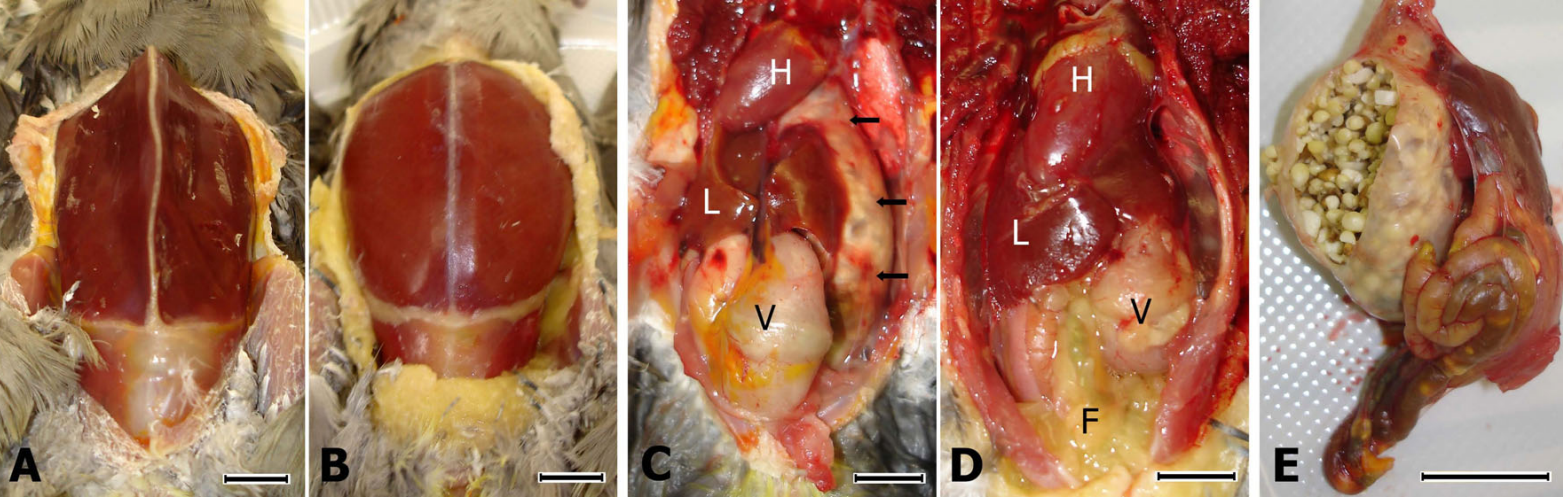
**Macroscopic pathological findings in an ABV4-inoculated cockatiel**. Bar size = 1 cm; F = peritoneal fat; H = heart; L = right liver lobe; V = ventriculus; A) Markedly reduced pectoral muscle mass and subcutaneous fat stores are clearly seen in an ABV4-inoculated cockatiel (cockatiel 3). B) Normal pectoral muscle mass and subcutaneous fat stores in a control bird (cockatiel 5). C) The peritoneal cavity of cockatiel 3, showing a severely distended and thin-walled proventriculus (arrows) that is visible well beyond the left liver lobe. The ventriculus is mildly distended and the peritoneal fat is dramatically reduced. D) In cockatiel 5, the proventriculus is of normal size, and; therefore, completely hidden behind the left liver lobe. Note the abundant peritoneal fat stores. E) The proventriculus and intestine of cockatiel 3. The thin wall has been cut, exposing a large amount of undigested seeds. Whole seeds are also visible through the intestinal wall.

In accordance with the clinical signs, on necropsy cockatiels 1 and 3 showed severe pectoral muscle atrophy and complete absence of peritoneal fat stores (Figure 2a). In contrast, cockatiels 2, 4, and 5 had normal pectoral muscle mass and extensive fat stores (Figure 2b). All three of the ABV-inoculated birds (cockatiels 1–3) showed dilatation of the proventriculus and to a lesser extent also of the ventriculus (compare Figure 2c and 2d). These findings were dramatic in cockatiels 1 and 3, where the giant thin-walled proventriculus was impacted with undigested seeds (Figure 2e). Whole seeds were also present throughout the intestine of these birds, and their livers appeared small and pale, consistent with chronic nutrient malabsorption (Figure 2c).

Moderate but clear distension of the proventriculus and also the proventriculus/ventriculs transitional area (isthmus) was observed in cockatiel 2; however, no proventricular impaction was present, the liver appeared normal, and undigested seeds were not present along the bird's intestine.

### Histopathology

**Figure 3:**
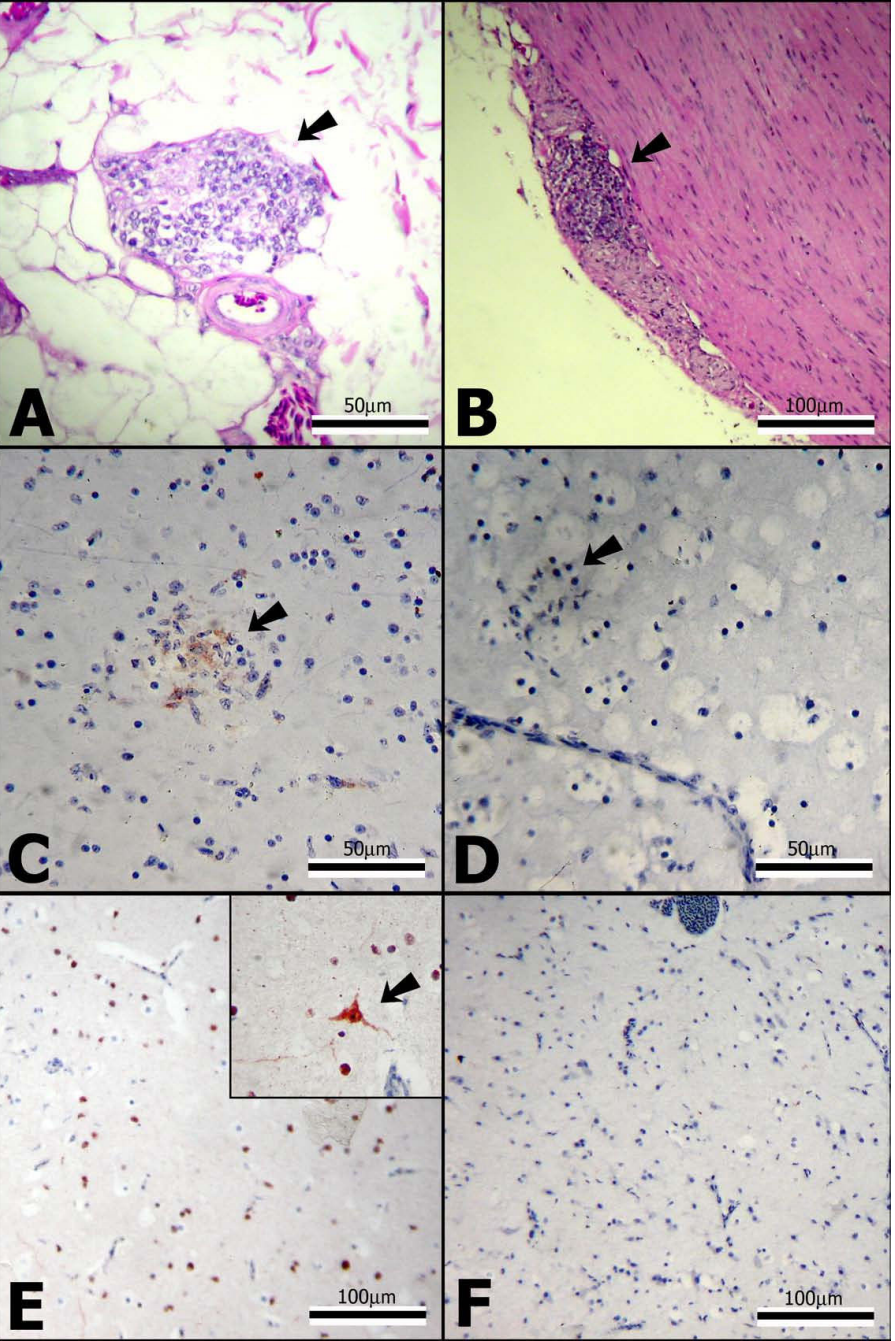
**Histological and immunohistochemical findings in ABV4-inoculated cockatiels**. A) Myenteric ganglioneuritis (arrow) in the crop of cockatiel 2 (hematoxyline and eosin [H&E] staining); B) Myenteric ganglioneuritis (arrow) in the ventriculus of cockatiel 3 (H&E staining). C) Positive IHC staining for ABVN associated with focal gliosis (arrow) in the cerebrum of cockatiel 1. Here, the staining appears to be mainly extracellular. D) For negative control, a section parallel to that in C was stained, using pre-immune rabbit serum instead of the anti-ABVN antibody. E) Widespread positive IHC staining for ABVN of neurons and glial cells of the cerebrum of cockatiel 2. At greater magnification, staining of a large neuron and its dendrites is shown (inset). F) For negative control, a section parallel to that in E was stained, using pre-immune rabbit serum instead of the anti-ABVN antibody.

Histopathologic lesions consistent with PDD were undetectable in the control inoculees. In contrast, all three of the ABV-inoculated cockatiels exhibited the PDD hallmarks of lymphoplasmacytic infiltrates within myenteric ganglia of the upper and middle GI tract (Figure 3). Interestingly, the most severely affected organ was the ventriculus, and in cockatiels 1 and 2 lymphoplasmacytic infiltrates were not limited only to ventricular nerves, but were also scattered throughout the thick tunica muscularis. This latter finding is not seen in all PDD cases, but was present in the bird from which the inoculum was prepared.

Beyond these general observations, a number of additional lesions were detectable in the ABV-treated birds. In cockatiel 1, lymphoplasmacytic infiltrates were present in the epicardium, epicardial ganglia, and one peri-adrenal ganglion, while in the cerebral grey matter of this bird multiple foci of gliosis, encircling small particles of amorphous eosinophilic material, were present along with mild lymphoplasmacytic perivascular cuffing. Similar histological findings were present in cockatiels 2 and 3, but with some differences. For example, in cockatiel-2 brain lesions were undetectable but marked lymphoplasmacytic perivascular cuffing was present in a section of the lumbosacral spinal cord. In cockatiel-3 mild lymphoplasmacytic perivascular cuffing was present in the cerebrum, but without gliosis. A detailed account of the histopathology results is provided in Additional File 1.

### Transmission electron microscopy screening of the inoculum and brain tissue from the study birds

**Figure 4:**
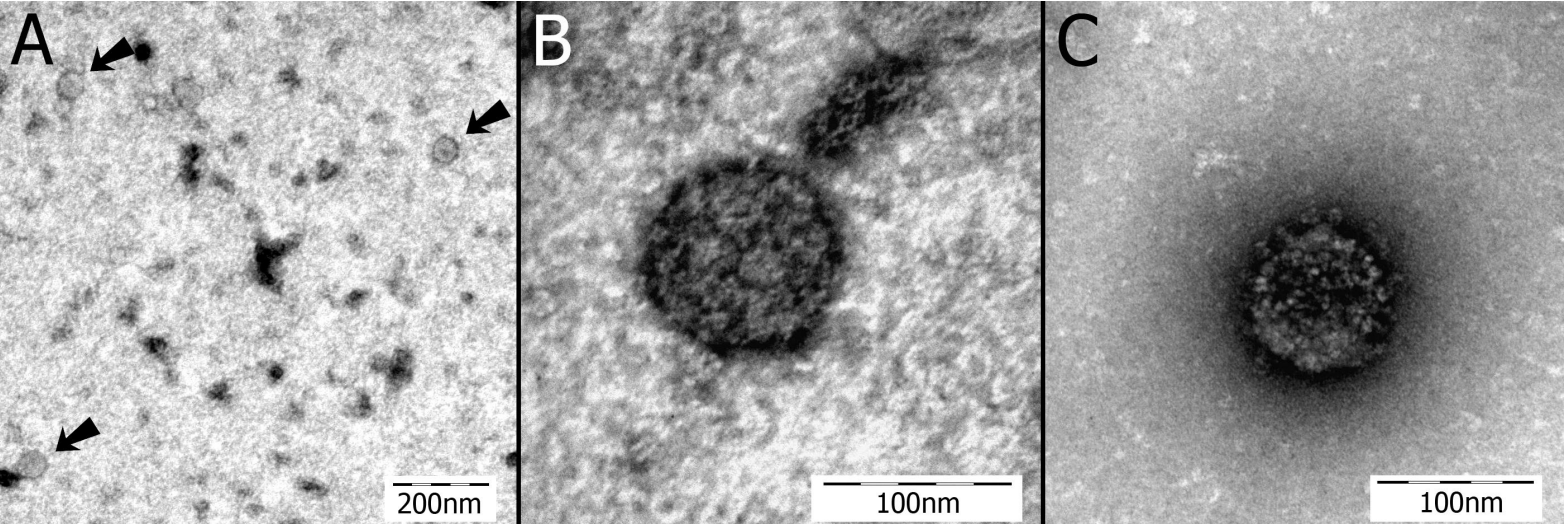
**Transmission electron microscopy images**. A) Brain homogenate from an ABV4(+) PDD(+) African grey parrot (the inoculum used in this study). Three spherical virus-like particles approximately 60 nm in diameter are shown [arrows] (negative staining with uranyl acetate). B) A virus-like particle from the same specimen in "A" shown at greater magnification. This particle is 98 nm in diameter (negative staining with uranyl acetate). C) A virus-like particle from the brain of cockatiel 1. This particle is 99 nm in diameter and is showing bold projections on its circumference (negative staining with uranyl acetate).

The original brain homogenate used for the inoculum as well as brain homogenates from the five study cockatiels were prepared for transmission electron microscopy (TEM) screening. Variable numbers of spherical virus-like particles 50–130 nm in diameter were present in brain homogenates of all ABV-inoculated birds and the original inoculum. Similar particles were not detected in the controls. Consistent with previously reported morphology of bornaviruses [12], these particles appeared to be surrounded by a membrane, and in some cases filamentous structures akin to glycoproteins, appeared to protrude from the membrane (Figure 4).

### Pyrosequencing of inoculum and analysis of ABV transmission

**Figure 5:**
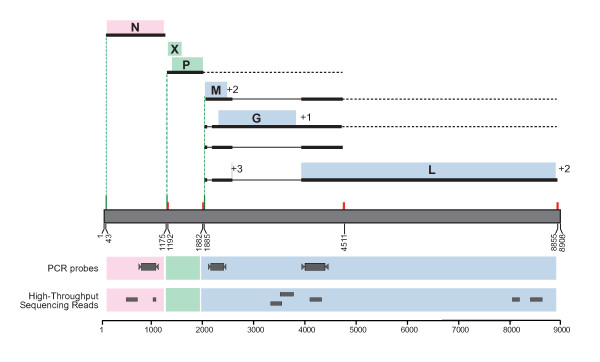
**Recovery of sequences matching ABV from the inoculum by high throughput pyrosequencing and RT-PCR**. The location of seven unique RNA sequences, recovered from the inoculum by high throughput pyrosequencing, is shown. These sequences share 73–100% sequence identity with existing ABV sequences (Additional file 2). In addition, the location of the RT-PCR primers for the N, M, and genes is shown, all of which yielded products that were consistent with ABV4 genome.

High throughput pyrosequencing was applied to the ABV4(+) brain homogenate to survey the diversity of viral species that might be present in the inoculum. From a total of 239,556 reads, we identified a set of 7 unique reads sharing 73–100% nucleotide sequence identity with existing ABV sequences (Figure 5, Additional file 2). Twenty-four reads that shared 40–89% sequence identity at only the amino acid level with viruses in the *Retroviridae *and *Astroviridae *families (Additional file 2) were also detected. An additional set of reads matched sequences derived from host (n = 223,081) and non-viral sequences in NCBI (n = 480). The remaining reads yielded no match to sequences present in NCBI (n = 15,803).

To determine if the ABV4 present in the inoculum was transmitted and correlated with PDD signs and symptoms, we performed blinded RT-PCR for the ABV N gene (ABVN) on all RNA samples taken over the course of the study. After unmasking the identity of the samples, we found that we detected ABVN RNA only in the ABV-inoculated birds (Table 1; Additional File 3). Independent RT-PCR for host RNAs on a set of matched tissue samples from ABV-inoculated and control birds confirmed the presence of intact RNA in the samples under study. Recovery of additional ABV RT-PCR products from the M and L gene provided further supporting evidence for ABV virus transmission only in the experimental birds (data not shown). Sequence analysis of these RT-PCR products revealed that the ABV recovered from the inoculees shared 99–100% sequence identity with the ABV4 sequences present in the original inoculum.

**Table 1:** Detection of ABV RNA in matched tissues of inoculees ^a^

	Experimentals (n = 3)	Controls (n = 2)
Brain	3/3	0/2
Spinal cord	1/3	0/1^b^
Adrenal	0/3	0/1^b^
Stomachs	2/3	0/2
Liver	0/3	0/2
Lung	0/3	0/2
Heart	1/3	0/2
Pectoral muscle	0/3	0/2
skin	0/3	0/2

^a ^RT-PCR for ABVN RNA and control RT-PCRs were performed for each specimen shown. ^b ^Control RT-PCRs were negative in one of the tissue samples from the control inoculees so the results for this bird were excluded from the analysis.

To assess the specificity of the correlation between the PDD symptoms and ABV transmission, we also performed independent RT-PCRs for the highly represented sequences for which we found detectable sequence similarity to *Retroviridae *and *Astroviridae *sequences (Additional file 4). Although RT-PCR products corresponding to these species were detectable in the original inoculum, none were recovered from the matched tissue specimens tested in the experimental or control inoculees.

### Tissue distribution and localization of ABV detected in inoculees

Upon detection of transmission of ABV infection and PDD signs and symptoms in the ABV-inoculees, we next investigated the viremia, viral shedding, tissue distribution and subcellular localization of ABV4 by RT-PCR for ABVN RNA and immuno-histochemistry for ABVN protein. By RT-PCR, none of the blood samples collected during the study tested positive for the presence of ABV RNA. This was also the case for all the cloacal and choanal swab samples for cockatiel 1 (the first bird to develop clinical signs of PDD). In contrast, a single choanal swab collected from cockatiel 3 (the second bird to develop clinical PDD) tested ABV positive on day 85 PI, and both choanal and cloacal swabs collected from cockatiel 2 (ABV-inoculated, but subclinical bird) tested ABV-positive on days 85 PI and 91 PI. From the tissues collected at necropsy, the brain, lumbosacral spinal cord, kidney and small intestine of cockatiel 1, the heart, spleen, pancreas, proventriculus, small intestine, and brain of cockatiel 2, and the brain, pancreas, small intestine, and proventriculus of cockatiel 3, tested positive for ABV RNA by RT-PCR. For a complete list of the specimens tested by RT-PCR, see Additional File 3.

By immunohistochemistry (IHC) for the ABVN protein, the brains of all 3 ABV-inoculated cockatiels were positive (Figure 3). In birds 2 and 3, ABVN staining was widespread throughout the cerebrum, localizing to the nuclei of neurons and glial cells. Staining of the cytoplasm was also present, but to a lesser extent. In contrast, in bird 1 there appeared to be N protein staining associated with areas of gliosis; in those foci staining appeared to be extracellular (Figure 3). ABVN-IHC staining was also observed in a single neuron of one affected myenteric ganglion of cockatiel 1, and in the lumbrosacral spinal cord of cockatiel 2, but was not detected in blood cells (including lymphocytes within perivascular cuffs), endothelial cells, connective tissue and other mesenchymal cell types. Epithelial involvement was difficult to determine due to the presence of non-specific ABVN-IHC staining in many of the sections. None of the tissues from the control birds stained positively for ABVN.

## Discussion

Our recent discovery of ABVs and their significant association with PDD [9] as well as the results of two other independent studies [10,11] offer for the first time in over 30 years a compelling etiological candidate for this disease. Here, we have successfully reproduced the clinical and pathological changes typical of PDD in cockatiels inoculated with brain homogenates containing ABV4. Our strategy for this study represents a modification of Koch's postulates, where high throughput pyrosequencing was used to obtain in-depth information on potential virus candidates in the inoculum, and this information was followed up by PCR testing of the study birds for all suspected viruses. This strategy may be of use in cases where virus isolation is difficult, or its methods are still under development, as was the case for ABV. Although we found viral sequences showing variable degree of homology to known genomes within the *Retroviridae *and *Astroviridae *families that were detected in the ABV(+) brain homogenates, none of these sequences were detected in tissues of the inoculated birds, making their role in PDD pathogenesis unlikely.

### Experimental ABV inoculation strategy

The inoculum used for this study was prepared from the brain of a PDD(+) ABV4(+) African gray parrot. We chose to use only brain tissue for an inoculum source, as it is easy to collect aseptically, should be free of the potentially contaminating flora of other body systems (e.g. GI flora), and is known to be a major target site of ABV [[10,11]; Kistler AL, unpublished data]. For inoculees, we utilized cockatiels because they were readily available and have been previously used for PDD research [13,14]. A combined intramuscular-oral-conjunctival-intranasal inoculation route was employed since the natural infection route of ABV is not yet known and a similar method was previously used to reproduce PDD, using an uncharacterized mixed tissue homogenate from a PDD(+) bird [14].

### Disease conferred by experimental inoculation with ABV(+) brain homogenate

All 3 cockatiels in our study that were inoculated with the ABV4(+) brain homogenate developed pathological lesions typical of PDD. Two of the three also showed overt clinical signs of PDD. Based on their weight loss patterns (Figure 1), the symptom-free or incubation period was 21 days in one bird and 31 days in the other, although for one cockatiel abnormal behavior (reduced ambulation, feather picking) was seen as early as day 9 PI. These birds reached the advanced stages of PDD at 64 and 95 days PI, respectively, showing a relatively slow progression of the disease. The third bird was symptom-free on day 95 PI despite suffering from moderate distension of the proventriculus. It is likely that this bird would have also eventually developed clinical PDD, but may have taken longer to do so. This spectrum of clinical findings is consistent with what is seen in naturally infected psittacine birds [3], and underline the great difficulty of identifying sick birds and preventing introduction of PDD into naïve collections.

The ABV-inoculated cockatiels in this study developed mainly lesions of the GI tract, with the most severe lesions being in the ventriculus. Brain lesions were mild or completely absent, and the clinical signs were those of the GI form of PDD. These findings are very similar to those seen in the original bird from which the inoculum was prepared, and may therefore reflect the role of the ABV strain type in determining lesion distribution patterns and the clinical manifestation of PDD. Differences in pathology and virulence of different ABV strains have not been studied to date, and warrant further experimental investigation.

The mechanisms by which PDD is induced remains unclear; however, based on the type of lesions seen, an immune-mediated pathogenesis is likely. In Borna disease, an immune mediated mechanism has long been proposed [15], and new evidence shows that this may also be the case for PDD. In a very recent study, it has been shown that anti-ganglioside antibodies were present in the serum of 98% of PDD cases compared with only 15.5% of the controls [16]. Furthermore, PDD could be induced by inoculating cockatiels with purified gangliosides [17]. These findings together with the discovery of the ABV-PDD association offer important clues to the mechanisms by which ABV may cause PDD. Further investigation of this intriguing topic is needed.

### ABV tropism detected in inoculees

A variety of tissues of the ABV4-inoculated cockatiels were ABV4 RNA(+) by RT-PCR. Of these, the brain, spinal cord and GI tract, were most commonly represented. IHC staining showed the presence of ABVN in nuclei and to a lesser extent also in the cytoplasm of neurons and glial cells of the brain and lumbosacral spinal cord. In other tissues the IHC results were more difficult to interpret, as epithelial tissues often showed non-specific staining. However, a recent independent study demonstrated broad tissue and cell tropism of ABV in PDD cases by RT-PCR and IHC with cross-reacting polyclonal antisera raised against the Borna disease virus P protein [11].

### Antemortem ABV-RNA detection in inoculees

In mammals, the initial site of bornavirus infection is thought to be the upper respiratory tract [18,19]. Moreover, PDD has been proposed to be transmitted via the oral-fecal route [3]. Thus, we obtained weekly oronasal and cloacal swab specimens from the experimentally inoculated birds to probe for the presence of ABV. In the experimentally inoculated birds, we detected ABV in choanal and cloacal specimens no earlier than day 85 PI, while viremia could not be detected at all during the study period. In contrast, the majority of choanal and cloacal specimens as well as 18% of blood samples collected during the same period from an asymptomatic cockatiel naturally infected with ABV2 (see Methods), tested positive for ABV2-RNA. One potential explanation for this finding is that ABV shedding may be limited in symptomatic PDD cases by the host's immune response (seen as lymphoplasmacytic infiltrates). Alternatively, the route of experimental inoculation used and/or other conditions present in this study may have resulted in reduced or variable ABV-RNA shedding compared with the naturally infected bird. Finally, the different ABV strains in the inoculees (ABV4) and the naturally infected cockatiel (ABV2) may account for this observation. Antemortem diagnosis of ABV infection and any factors that may affect it are of great clinical and epidemiological importance, and should be at high priority for further investigation.

The detection of a naturally occurring clinically asymptomatic ABV2-infected cockatiel at the start of this study is of particular interest in light of the results mentioned. To date (9 months after its purchase), this bird remains symptom-free, and although it may still develop PDD in the future, it is also possible that this cockatiel is not susceptible to this particular virus strain, or that this ABV strain is of low pathogenicity in general, or specifically in cockatiels. This observation raises the possibility that ABV infections may not always confer clinically overt signs of PDD, and long-term asymptomatic carriers may play a role in the epidemiology of ABV. Further investigation of outcomes associated with both naturally and experimentally ABV-infected psittacines are required to better understand these findings.

## Conclusion

Here, we present, for the first time, the results of experimental inoculation of a psittacine species with ABV. We found that experimental inoculation of naïve cockatiels with ABV4(+) brain homogenate produced classical PDD in all inoculees. These findings together with those previously reporting a statistically significant association between ABV and PDD [9], provide compelling evidence that ABV infection can confer PDD in psittacines. The detection of both experimentally and naturally occurring ABV infections with moderate to no overt clinical signs of PDD raises the possibility that variability in ABV strain virulence, host response and/or the interplay of these factors may influence the development and transmission of PDD in psittacines, warranting further investigation into the prevalence of clinically symptomatic and asymptomatic ABV infection in captive as well as free ranging avian species.

## Methods

### Inoculation experiment

The inoculation experiment was approved by the animal care committee at Kimron Veterinary Institute (KVI), Bet Dagan, Israel. For the experiment, six male wild-type cockatiel parrots were purchased from a local breeder. The birds were determined to be in good health based on physical examination, complete blood-cell count and fecal cytology. To be included in this study, the cockatiels had to be ABV(-) by RT-PCR, and show no histological lesions suggestive of PDD in multiple crop biopsy sections. To test for pre-existing ABV-infection, whole blood as well as cloacal and choanal swabs were collected from each bird, submersed in an RNA preservative (RNAlater; Qiagen, Valencia, CA), and kept frozen at -80°C. In addition, full thickness crop biopsies of about 10 mm in diameter were surgically collected from all birds as previously described [9]. Approximately one fourth of each biopsy was submersed in RNAlater and frozen at -80°C until RT-PCR testing, while the rest was placed in 10% neutral buffered formalin, sectioned into 4–6 slices, and prepared for histopathological examination. The crop biopsy and several choanal and cloacal swabs of the sixth bird tested positive for ABV2 by RT-PCR. This bird was therefore removed from the inoculation study; however, we continued to monitor it for ABV-RNA shedding for the duration of the study period.

The cockatiels were housed in individual cages and placed in animal isolation units, where they were allowed to recover from surgery and acclimatize for 8 days prior to inoculation. Drinking water and a commercial seed-based diet were provided on an ad lib basis, and ambient temperature was kept at 28°C.

The inoculum was prepared from brain tissue of an African grey parrot (*Psittacus erithacus*) that had shown classical gastrointestinal signs of PDD prior to death (KVI# F45b). The bird was confirmed to be PDD(+) by histology and ABV4(+) by RT-PCR and subsequent sequencing. Approximately 1 g tissue was macerated by two passages through a 2.5 ml syringe and was then diluted 1:4 in sterile saline. The preparation underwent two 24 h freeze-thaw cycles at -80°C, before centrifugation at 4°C at 4000 × g for 10 min. The supernatant was collected and kept on ice until use (within 90 min). This same methodology was used to prepare a sham inoculum from brain tissue of an African grey parrot that had died from causes other than PDD, and was ABV(-) by RT-PCR (KVI# F27b).

To test for the presence of bacteria in the inoculum, routine microbial culture was attempted on blood-agar and McConkey's agar media, while high throughput pyrosequencing was employed to test the inoculum for the presence of viral RNA (see below). In addition, TEM was used to screen the inoculum for the presence of viral particles.

Birds included in the study were randomly assigned to one of two treatment groups. Three male cockatiels were inoculated with the ABV-containing homogenate, while the other two males received the sham inoculum. For inoculation/sham inoculation, a combined intramuscular (0.2 ml injected by 28 G needle into the left pectoral muscle), oral (0.2 ml), intranasal (1 drop in each nostril), and conjunctival (one drop on each eye) route was used. After inoculation, the cockatiels were monitored for 95 days by an observer who was blinded to their treatment status. This included daily observation of the birds' general attitude, behavior, gait, feeding activity and uro-fecal output. BW was recorded weekly using an electronic scale. Whole blood, choanal and cloacal swabs were collected on days 1, 2, 4, 8, 11, 13, 21, 26, 35, 40, 57, 63, 70, 77, 85, 93 PI. All samples were immersed in RNAlater and frozen at -80°C. Birds that lost >25% BW and/or showed signs of advanced disease (e.g. marked lethargy, weakness, neurological signs) during the study were humanely euthanized by CO_2 _inhalation. All other birds were humanely euthanized at the end of the study. Diagnostic necropsies were performed for all birds under a biohazard hood, using aseptic technique. For each bird, a complete set of tissue samples was collected in RNAlater and frozen at -80°C for RT-PCR testing. A second set of tissue samples was placed in 10% neutral buffered formalin for histopathology. In addition, tissue samples of brain and proventriculus were collected for TEM, and stored frozen at -80°C with no additive.

### ABV nucleocapsid gene cloning, expression and polyclonal antibody generation

The open reading frame (ORF) encoding the ABV nucleocapsid (ABVN) gene flanked with BamHI and NotI restriction sites was amplified from ABV2 total RNA [9] by RT-PCR with the following primers: ABVN-BamHI, 5'GCGCGCCCCC**GGATCC***ATGCCACCCAAAAGGCAAAG*-3' and ABVN-NotI, 5'-GCGTGCTACGCCAT**GCGGCCGC***CGTTTGCAAATCCAGTTACGCC*-3' (restriction sites bolded, ABVN ORF overlap italicized). The resulting product was sequence-confirmed, digested with BamH1 and NotI and subcloned into a BamHI/NotI-digested modified pMAL vector (gift from Matthew C. Good, UCSF), which contains a 6xHis tag on the C-terminus (His_6_), and a maltose-binding protein (MBP) tag on the N-terminus. Ligation into this vector generated a TEV protease cleavage site (tev) between the N-terminal MBP tag and ABVN ORF. The sequence-confirmed, modified pMAL vector containing the ABVN ORF was transformed into pRIL+ BL21(DE3) E. coli and recombinant MBP-tev-ABVN-His_6 _protein expression was induced with 250 uM IPTG at 37°C for 4 hours. Cells were lysed in 50 mM Tris pH 8.0, 100 mM NaCl, and 1× Roche Complete Protease Inhibitors (Roche Applied Science; Indianapolis, IN) using 3 cycles through a microfluidizer. MBP-tev-ABVN-His_6 _protein was purified from cell lysates via Ni-NTA column chromatography followed by amylose column binding and elution with maltose. The resulting eluate was concentrated with a 50 kDa Amicon Ultra (Millipore; Billerica, MA) and incubated with 10 units of TEV protease for 1 day at 4°C. The cleavage reaction mixture was then diluted into 25 mM Tris, pH 7.0, 100 mM NaCl buffer and loaded on a 1 mL RESOURCE S column (GE LifeSciences, Piscataway NJ) to separate the cleaved MBP tag and TEV protease from the ABVN- His_6 _protein by ion exchange chromatography. Resulting fractions containing ABVN-His_6 _protein were combined, concentrated using a 15 kDa Amicon Ultra, and further purified based on size using a 24 mL Superdex200 column (GE LifeSciences). Fractions containing purified ABVN-His_6 _were combined and concentrated using a 15 kDa Amicon Ultra. 2.5 mg of this purified ABVN-His_6 _was used for polyclonal antibody generation in rabbits (Pacific Immunology; Ramona, CA).

### Histopathology and immunohistochemical staining

Tissue specimens were processed for routine histopathology, sectioned at 6 μm, and stained with hematoxylin and eosin. To increase the sensitivity of PDD-specific lesion detection, multiple sections (3–5) were prepared for the crop, ventriculus and proventriculus of each bird. Crop biopsies were sectioned 5–6 times. For each bird, a second set of slides was prepared for immunohistochemical staining. Briefly, tissue sections underwent deparaffinization and rehydration, followed by treatment with 3% H_2_O_2 _for 10 min. The sections were then washed twice with PBS, and incubated for 60 min at room temperature with rabbit anti-ABVN polyclonal antibody (see above) at 1:500 or 1:1000 dilution. After rinsing in PBS for 5 min, horseradish peroxidase polymer-conjugated anti-rabbit IgG (Jackson ImmunoResearch Laboratories, Inc., West Grove, PA) was added for 30 min. The sections were again rinsed with PBS, and the substrate-chromogen solution (Zymed AEC; San Francisco, CA) was added for 3 min at room temperature. Specimens were then rinsed, counterstained with hematoxylin, and allowed to dry at 60°C for 2 h.

### Transmission electron microscopy

Frozen tissue specimens (-80°C; no additive) were allowed to thaw at room temperature and were then minced with a scalpel blade or macerated by 2 passages through a sterile 2.5 ml syringe. Approximately 1 g of each specimen was placed in a test tube containing 4 ml PBS followed by vortexing. The preparations were centrifuged at 4000 g at 4°C for 10 min, and the clarified supernatant was collected. Virus concentration was then attempted by ultra-centrifugation (Kubota 7800; Kubota, Japan) at 40,000 g for 5 h, followed by discarding approximately 95% of the supernatant and re-suspending the sediment in the remaining fluid. End products were then stored at -80°C until use.

For negative staining examination, carbon-stabilized and Formvar-coated 300-mesh copper grids were used. The grids were floated on a drop of suspect sample and allowed to adhere to the drop for 2 min at room temperature. The grid was then removed and excess liquid was drained by blotting the edge of the grid with filter paper. Next, the grid was floated on a drop of 2% aqueous uranyl acetate solution for 30 sec. The excess stain was removed as before and the specimens were examined by TEM, using a Tecnai G2 Spirit electron microscope (FEI company, Hillsboro, OR).

### RNA extraction

For RNA extractions from tissue and whole blood, specimens underwent two 24 h freeze-thaw cycles at -80°C followed by scalpel mincing (tissues only). Total RNA was then extracted using the TRI Reagent^® ^kit (Molecular Research Center, Cincinnati, OH), following the manufacturer's instructions. RNA extractions from choanal and cloacal swabs were performed by the QIAamp viral RNA kit (Qiagen, Valencia, CA). The end product was lyophilized and stored at -80°C until testing.

### Pyrosequencing and analysis of RNA extracted from the challenge inoculum

Five independent random amplification reactions generated from approximately 50 ng of total RNA derived from the ABV4(+) brain homogenate were pooled together for library generation for pyrosequencing using standard 454/Roche GS-FLX protocols [20]. After filtering primer sequences, exact duplicates, low complexity sequences and reads < 36 bp long, a working set of 239,556 reads remained for analysis. To filter reads potentially derived from psittacine host tissue RNAs, the working set of reads was aligned to a database of all Aves sequences extracted from NCBI (n = 918,511) using megablast (e = 10^-10^; word size = 12), followed by progressively lower stringencies (down to e = 10^-4^; word size = 12). The remaining 16,475 Aves-filtered reads were next aligned to all sequences in NCBI to identify viral and non-viral sequences via a high stringency megablast (e = 10^-10^; word size = 12) followed by a lower stringency blastn (e = 10^-6^; word size = 8), and blastx (e = 10^-6^; word size = 4) were performed. A final low stringency tblastx alignment (e = 10^-3^; word size = 3) to a database containing all viral sequences present in NCBI was performed to screen for potential divergent viral species missed in the prior screens. Candidate viral reads identified from this final screen were verified by re-blasting against all NCBI sequences. Reads that failed to yield viral sequence matches in this final re-blast were considered false positives and discarded as potential viral sequence. Reads that did yield viral sequences in the re-blast against NCBI were considered candidate viral sequences and were grouped according to viral species, aligned to identify regions of overlap useful for RT-PCR primer design.

### RT-PCR for ABV RNA detection

Initial RT-PCR for ABV was performed in a blinded fashion on all RNA samples extracted for the challenge study. Each sample was used as input template for 1-step RT-PCR assay (Qiagen, USA, Valencia CA) using previously described primers [9] for amplification of ABVN, ABVM and ABVL RNAs. Resulting RT-PCR products were gel purified, incubated with 0.25 mM dATP and recombinant Taq polymerase (Invitrogen, Inc., Carlsbad CA, USA) at 72°C for 15 minutes, then subcloned into the pCR2.1 TOPO T/A cloning vector (Invitrogen, Inc., Carlsbad CA, USA). For each subcloned RT-PCR product, 3 independent transformants were amplified and sequenced using M13 forward and M13 reverse primers. Upon sequence confirmation of the identity of RT-PCR products, sample identities were unmasked and a follow-up control 1-step RT-PCR assay using primers directed against a highly conserved region of 18s rRNA sequences detected from the initial brain inoculum in 124 overlapping reads (rRNAF: 5'-CGGCGTCCAAC-TTCTTAGAG-3', rRNAR: 5'-AATGGGGTTCAACGGGTTAC-3') was performed on tissue-matched case and control RNAs. For all 1-step RT-PCR assays described above, 5 uL of template RNA was used in a final reaction volume of 25 uL, incubated at 50°C for 30 minutes, followed by a 95°C incubation for 15 minutes, and 35 cycles of denaturation at 94°C for 30 seconds, annealing at 50°C for 30 seconds, and elongation at 72°C for 30 seconds.

### RT-PCR screening for non-ABV viral species detected in the inoculum

Primers for RT-PCR recovery of viruses other than ABV were designed based on candidate viral sequences recovered in the pyrosequencing analysis of the brain inoculum (Additional file 4). RT-PCR for each of these viral species was performed on RNA derived from matched tissues specimens from each bird to screen for the presence of these viruses in both inoculated and control birds.

## List of abbreviations

ABV: avian bornavirus; ABV2: avian bornavirus 2; ABV4: avian bornavirus 4; PDD: proventricular dilation disease; GI: gastrointestinal; BW: body-weight; PI: post-inoculation; TEM: transmission electron microscopy; IHC: immunohistochemistry; ORF: open reading frame; ABVN: ABV nucleocapsid; His_6_: 6xHis tag; MBP: maltose-binding protein.

## Competing interests

Authors AYG, ALK, AG, SC, JLD, DG and AL are inventors on a patent application which describes applications of methods and results presented herein and in a related study [9]. The IP rights are co-owned by the Regents of the University of California, the Lahser Interspecies Research Foundation, Ady Gancz, and the Kimron Veterinary Institute.

## Authors' contributions

AYG participated in the conception, design, and coordination of the study, performed the inoculation of the cockatiels, participated in the clinical monitoring of the inoculees, performed necropsies, participated in the TEM screening of samples, and, together with ALK, wrote the manuscript; ALK participated in the design and coordination of the study, performed RT-PCR screening and sequence analysis of RNA samples, performed the pyrosequencing and analysis of RNA extracted from the inoculum, and, together with AYG, wrote the manuscript; AG developed the anti-ABVN antibodies used for IHC, and helped revise the manuscript; YF participated in the inoculation experiment, in specimen collection and performance of necropsies, in RNA extractions from specimens, and performed RT-PCR assays; SM participated in specimen collection and monitoring of the inoculees, and performed the bacteriological assays; SP supervised the histological and IHC evaluation of specimens in this study; AB coordinated the processing of samples for histology and IHC, and participated in their screening; NP participated in the inoculation and clinical monitoring of the cockatiels, collected clinical specimens from the cockatiels, extracted RNA, and assisted in specimen processing for IHC; SC participated in the design of the study, and helped revise the manuscript; JLD and DG participated in the conception and design of the project, supervised its execution (UCSF part), and helped revise the manuscript; AL participated in the conception and design of the project, supervised its execution (KVI part), participated in the inoculation experiment, specimen collection, TEM screening of specimens, and helped revise the manuscript. All authors read and approved the final manuscript.

## Supplementary Material

Additional file 1 **Histology results**. This file provides a detailed account of the histopathological findings in tissues of the study birds. Click here for file

Additional file 2 **Viral RNA sequences recovered from the inoculum by highthroughput pyrosequencing**. The file provides details on 31 RNA sequences that were recovered from the inoculum, and that match members within the *Bornaviridae*, *Retroviridae*, and *Astroviridae *families. Click here for file

Additional file 3 **RT-PCR results for ABVN RNA**. The file lists all the specimens tested by RT-PCR for ABVN RNA and their status. Click here for file

Additional file 4 **Primers used RT-PCR screening for retroviral and astroviral RNA**. The file contains the sequence information of the primers used to screen tissues of the study birds for retroviral and astroviral RNA present in the inoculum. Click here for file
