# Supplementary material for: Experimental induction of proventricular dilatation disease in cockatiels (Nymphicus hollandicus) inoculated with brain homogenates containing avian bornavirus 4
Source: Virol J. 2009 Jul 9;6:100. doi: 10.1186/1743-422X-6-100 (PMC2717941; doi:10.1186/1743-422X-6-100)
Supplement: Additional file 2 — Viral RNA sequences recovered from the inoculum by highthroughput pyrosequencing. The file provides details on 31 RNA sequences that were recovered from the inoculum, and that match members within the Bornaviridae, Retroviridae, and Astroviridae families. [file 1743-422X-6-100-S2.pdf]

Viral RNA sequences recovered from the inoculum by high throughput pyrosequencing

Table 1 - Reads from inoculum matching avian bornavirus viral species

Read	Length	Viral species	NCBI identifier	Name	% identity*	Evalue	coverage†
356266_0898_0509	247	ABV	FJ169441.1	Bornavirus parrot/PDD/2008 strain 1034_1322	89	2.00E-46	0.18
458379_0620_1472	244	ABV	FJ169440.1	Bornavirus parrot/PDD/2008 strain 1367	94	6.00E-45	0.19
257206_0641_1258	240	ABV	FJ169441.1	Bornavirus parrot/PDD/2008 strain 1034_1322	100	3.00E-48	0.32
035286_1771_0635	140	ABV	EU781967.1	Borna disease virus isolate bil, complete genome	73	1.00E-29	0.46
331364_1639_3879	222	ABV	FJ169441.1	Bornavirus parrot/PDD/2008 strain 1034_1322	99	1.00E-42	0.33
071012_0389_2593	236	ABV	EU781967.1	Borna disease virus isolate bil, complete genome	90	2.00E-30	0.33
266025_0890_1915	60	ABV	FJ169440.1	Bornavirus parrot/PDD/2008 strain 1367	100	1.00E-06	0.33

*amino acid identity of highest scoring tblastx match
†fraction of read covered by alignment to higiest scoring tblastx match

Table S2 - Reads from inoculum matching retroviral species

Read	length	Viral species	NCBI identifier	Name	%identity*	Evalue	Coverage†
281412_0830_1095	241	Avian endogenous retrovirus	AJ236114.1	Sericulus bakeri retrovirus partial pol and pro genes isolate RV-Bower Bird II	89	8.00E-20	0.57
355379_0549_0128	251	Avian endogenous retrovirus	AY820084.1	Rhea Americana endogenous virus class II-related clone	72	2.00E-16	0.39
416898_1006_1566	180	Avian endogenous retrovirus	AY820100.1	Catharus guttatus endogenous virus class II-related clone	72	5.00E-08	0.53
172465_0910_2228	248	Avian endogenous retrovirus	AY820065.1	Apteryx Australia endogenous virus class II	60	2.00E-21	0.77
146837_1472_0407	231	Avian leukosis virus	M14898.1	ALVNY203A Avian leukosis virus strain NY203	67	4.00E-12	0.56
377534_1666_2595	235	Avian leukosis virus	AY608692.1	Avian leukosis virus envelope protein (env)	66	2.00E-11	0.84
152062_0175_1296	243	Avian leukosis virus	XR_026828.1	Predicted: Gallus gallus similar to gag protein	40	2.00E-04	0.81
444062_1714_0065	236	Avian leukosis virus-related	XM_001235407.1	Predicted: Gallus gallus similar to gag/env protein	65	1.00E-04	0.29
092749_0764_3453	245	Avian retrovirus-related	XM_00219940.1	Predicted: Taeniopygia guttata similar to Pro-Pol polyprotein	59	2.00E-27	0.97

398087_0492_1844	249	RV-Tinamou	AJ225235.1	RV-Tinamou partial mRNA for polyprotein	52	2.00E-06	0.58
055495_1630_3776	219	Avian retrovirus	AJ236126.1	Perdix perdix retrovirus partial pol and pro genes isolate RV-partridge	80	3.00E-06	0.36
055968_1321_1194	182	HIV/SIV-like	AF383261.1	HIV-1 98CMA010 from Cameroon envelope glycoprotein (env) gene	58	0.009	0.21
473627_1296_1228	179	HIV/SIV-like	M11841.1	SIVRV1CG Simian SRV-1 type D retrovirus (L47.1), complete genome	51	2.00E-08	0.82
537382_1249_0392	199	HIV/SIV-like	GENE ID: 100226068 LOC100226068	Similar to pol protein Taniopygia guttata	66	1.00E-08	0.59
010928_1475_1136		HIV/SIV-like	M11841.1	SIVRV1CG Simian SRV-1 type D retrovirus (L47.1) complete genome	53	7.00E-11	0.89
321868_1771_2307	251	HIV/SIV-like	M11841.1	SIVRV1CG Simian SRV-1 type D retrovirus (L47.1),complete genome	59	1.00E-19	0.76
233849_0660_0586	176	HIV/SIV-like	M11841.1	SRV1CG Simian SRV-1 type D retrovirus (L47.1), complete genome	52	7.00E-11	0.90
032321_1459_1257	230	HIV/SIV-like	U05079.1	Simian immunodeficiency	40	8.00E-04	0.35

				virus SIVRhE543 c.one 1-11 envelope glycoprotein			

*amino acid identity of highest scoring tblastx match
†fraction of read covered by alignment to higiest scoring tblastx match

Table S3 - Reads from inoculum matching Astroviridae viral species

Read	Length	Viral species	NCBI identifier	Name	% identity*	Evalue	coverage†
287362_0192_3673	229	Turkey astrovirus	EU143850.1	Turkey astrovirus strain TAstV/TX/00	41	0.009	0.511
094709_0461_2000	230	Turkey astrovirus	EU143850.1	Turkey astrovirus strain TAstV/TX/00	41	0.005	0.509
208816_0736_3748	233	Turkey astrovirus	EU143850.1	Turkey astrovirus strain TAstV/TX/00	41	0.005	0.502
186567_1000_1068	228	Turkey astrovirus	EU143850.1	Turkey astrovirus strain TAstV/TX/00	41	0.005	0.513
193911_1738_0676	229	Turkey astrovirus	EU143850.1	Turkey astrovirus strain TAstV/TX/00	41	0.005	0.511
351350_1441_3268	233	Turkey astrovirus	EU143850.1	Turkey astrovirus strain	41	0.005	0.502

				TAstV/TX/00			

*amino acid identity of highest scoring tblastx match

†fraction of read covered by alignment to higiest scoring tblastx match
