# Supplementary material for: Experimental induction of proventricular dilatation disease in cockatiels (Nymphicus hollandicus) inoculated with brain homogenates containing avian bornavirus 4
Source: Virol J. 2009 Jul 9;6:100. doi: 10.1186/1743-422X-6-100 (PMC2717941; doi:10.1186/1743-422X-6-100)
Supplement: Additional file 4 — Primers used RT-PCR screening for retroviral and astroviral RNA. The file contains the sequence information of the primers used to screen tissues of the study birds for retroviral and astroviral RNA present in the inoculum. [file 1743-422X-6-100-S4.doc]

Primers designed for detection of viruses identified in ABV(+) inoculum in case and control bird tissues

| **Viral target** | **Forward primer** | **Reverse primer** |
| --- | --- | --- |
| Endogenous retrovirus-related | CGTGCATGACTTTGGATGAG | CACCCAGTAGGACCCATCAG |
| Turkey astrovirus-related | CATCTGTCACCCAGAAGAGC | CCTGTAACATGGCACTGAGC |
| Avian leucosis virus-related | AACATTTGTGAAGAATATCAGTGG | GATAAAGTGCAACTTTTTGACACG |
| SIV/HIV-related | GATTTTTGCGAGGTTGTAAGC | TTGTATCCTTATACCACCAATCTTCC |
| *Perdix perdix* retrovirus-related | TGCAGTGGGTTGAATACCAA | CGGCCAGCAACAGAAGGTA |
|  |  |  |
| Retrovirus Tinamou-related | ATCAGGCAGTTTCCATGTCC | CCGCAAAGTGAAAGTTGTAGG |
|  |  |  |
| HERV-related | TCCATCCCTTTTCAGCTGTT | GCTCAGGGCTAAAGGGTTGT |
